# Advancing School‐Based Efforts to Reduce Trauma and Violence Influences on Youth

**DOI:** 10.1111/josh.70238

**Published:** 2026-09-29

**Authors:** Jerica Knox, Tiffany Beason, Sara B. Johnson

**Affiliations:** ^1^ School of Medicine, University of Maryland, National Center for School Mental Health Baltimore Maryland USA; ^2^ School of Medicine, Johns Hopkins University Baltimore Maryland USA; ^3^ Bloomberg School of Public Health, Johns Hopkins University Baltimore Maryland USA

**Keywords:** cultural responsiveness, school mental health, secondary traumatic stress, trauma and violence

## Abstract

**Background:**

Trauma and violence exposure are critical barriers to educational equity, affecting student mental health and academic achievement. Despite growing attention to trauma‐informed practices in schools, implementation remains uneven and often overlooks the complexity of trauma experiences or their systemic roots.

**Contributions to Theory:**

Grounded in and extending Basch's (2011) framework for prioritizing health conditions to reduce educational disparities, this paper synthesizes lessons learned and proposes a path forward for school‐based trauma prevention and intervention. We identify three key opportunities: (1) expanding school‐based trauma definitions to include chronic and identity‐based stressors such as racialized trauma and immigration‐related adversity; (2) leveraging comprehensive, school‐wide frameworks to support infrastructure development, cross‐sector partnerships, and sustainable implementation; and (3) elevating secondary traumatic stress as a core concern within trauma‐informed systems.

**Implications for School Health Policy, Practice, and Equity:**

Schools can use public health‐oriented frameworks to align trauma‐informed practices with equity goals. Key actions include conducting inclusive needs assessments, providing culturally responsive professional development, fostering cross‐sector partnerships, and embedding policies that support educator wellness and prevent secondary traumatic stress.

**Conclusions:**

By broadening definitions, centering equity, and supporting staff well‐being, schools can create trauma‐informed systems that are sustainable, responsive, and healing‐centered.

Childhood trauma refers to the psychological and physiological responses that occur when individuals experience events or conditions that threaten their physical or psychological well‐being, such as violence, abuse, or discrimination [1]. These responses can lead to alterations in physiology, physical and mental health, learning, and cognition [2, 3, 4]. As many as two thirds of US children have been exposed to one or more potentially traumatic events [5, 6, 7]. Individuals from racially minoritized groups and those experiencing poverty are disproportionately exposed to these adversities [7]. In addition, potentially traumatic experiences extend beyond home and community settings; some school‐aged children and adolescents encounter such experiences in school as a result of bullying and school violence [8, 9]. Students who report feeling unsafe at school have significantly lower standardized test scores than their peers, and those exposed to violence are more likely to be absent from school [8, 9]. For youth from racially minoritized groups, racial trauma in school is linked to lower grades, reduced engagement, and a higher likelihood of leaving school without graduating [10]. Together, these patterns underscore the critical role of trauma and violence exposure as barriers to both student health and educational equity.

Within this broader context, Basch proposed a framework in 2011 for implementing high‐quality, evidence‐based school health programs to address health‐related barriers that contribute to educational disparities [11]. Among the priority areas identified, Basch emphasized aggression and violence as critical factors that disproportionately affect racially minoritized and urban youth and interfere with learning. National data at the time illustrated the magnitude of the problem, with approximately 35% of high school students reporting involvement in physical fights and nearly 8% reporting being threatened or injured with a weapon at school in the prior year [11]. Although originally conceptualized in terms of observable behaviors such as fighting, bullying, and threats of violence, these experiences can also be understood as forms of trauma exposure that shape students' psychological and physiological responses and, in turn, their capacity to engage in school. The framework highlights the importance of coordinated, school‐based strategies and evidence‐based interventions to address these conditions and improve both health and academic outcomes.

Since Basch's call to action, growing recognition of the negative effects of trauma and violence has driven a broader shift in how schools conceptualize and address barriers to learning. Encouragingly, national surveillance data indicate that school crime and safety incidents, particularly physical fights and weapon carrying, have declined since 2012, while students' perceptions of school safety have improved [12, 13]. These trends suggest progress toward Basch's original priorities for reducing aggression and violence in school settings. Still, there is growing recognition that students' exposure to trauma, including but not limited to violence experienced at home, at school, or in their communities, poses a serious threat to student health and educational success. In addition, since 2011, schools have increasingly adopted trauma‐informed practices designed to recognize and respond to the influence of trauma on students' behavior, learning, and relationships. Twenty‐four of 50 US states now have policies or legislation requiring professional development on trauma‐informed practices for school personnel, reflecting rapid national adoption [14]. Evaluations of trauma‐informed practices in schools have shown improvements in educators' trauma knowledge and practice efficacy and students' socio‐emotional and behavioral outcomes [15]. Despite this progress, trauma prevention and intervention in schools remain unevenly implemented and under‐addressed.

In this paper, we synthesize key lessons learned in trauma and violence prevention and intervention in schools and extend Basch's [11] framework to more fully address trauma and violence exposure as critical barriers to student health and educational equity. We highlight three opportunities for strategic, coordinated approaches that can help mitigate disparities and promote safer, more supportive learning environments: (1) expanding the definition of trauma and violence exposure in schools to better reflect students' lived experiences; (2) leveraging schools to prevent and respond to students' trauma‐related needs through comprehensive, coordinated systems; and (3) addressing the needs of school staff to support effective and sustainable implementation of interventions to prevent and respond to student traumatic stress (Table 1).

**TABLE 1:** Opportunities and actionable strategies for trauma‐informed schools.

	Description	Actionable strategies
*Opportunity 1*: Expand the definition of trauma and violence exposure in schools	Broaden school‐based conceptualizations of trauma beyond discrete interpersonal events to include chronic, systemic, and identity‐based adversities	Integrate structural and identity‐based trauma into policies and training Use needs assessments to capture diverse trauma exposures Disaggregate data to identify inequities Align screening with expanded trauma definitions
*Opportunity 2*: Advance school‐based trauma prevention efforts	Strengthen coordinated, multitiered school mental health systems to deliver equitable trauma prevention and intervention.	Strengthen Tier 1 climate supports Expand Tier 2/3 evidence‐based and culturally responsive interventions Invest in workforce capacity (training, supervision, staffing) Build cross‐sector partnerships to expand access
*Opportunity 3*: Address secondary traumatic stress in trauma‐informed schools	Address secondary traumatic stress among school personnel to support effective and sustainable implementation.	Include secondary traumatic stress prevention in professional development Provide reflective supervision and peer support Embed staff wellness into systems and policies Monitor burnout, retention, and STS indicators

## 1 Opportunity 1: Expand the Definition of Trauma and Violence Exposure in Schools

When Basch identified aggression and violence as key health barriers to learning, school‐based efforts to address these issues largely focused on preventing or responding to discrete incidents of interpersonal harm—such as bullying, fighting, or exposure to violence. In the years that followed, these early prevention efforts contributed to the rise of trauma‐informed education, which sought to recognize and respond to the effects of trauma on student learning and behavior. However, most early frameworks conceptualized trauma narrowly as exposure to acute or catastrophic events, with limited attention to the systemic and identity‐based factors that also shape students' experiences. For example, the Trauma and Learning Policy Initiative [16], one of the earliest and most widely cited trauma‐informed school frameworks, defines trauma primarily through experiences such as abuse, neglect, or witnessing violence, reflecting an individual lens. This early conceptualization profoundly shaped the field, influencing how educators are trained and how schools continue to operationalize trauma‐informed approaches today. In a recent systematic narrative review of 24 studies examining content in trauma‐informed professional development in K–12 schools, nearly half (11/24) did not incorporate culturally responsive, anti‐racist, or equity content [15]. This pattern suggests that, while trauma‐informed education is growing, many school personnel are still being trained to conceptualize trauma primarily as an individual or interpersonal experience rather than one that can also stem from structural and identity‐based stressors such as racism or immigration‐related adversity. Notably, many programs cited the Substance Abuse and Mental Health Services Administration's (SAMHSA) six principles of trauma‐informed care, which include attention to cultural, historical, and gender issues, yet these principles were rarely translated into actionable training content or practice. This suggests that, even when frameworks acknowledge structural factors, implementation often remains focused on individual adversities. Ginwright's critique of traditional trauma frameworks underscores this limitation, noting that these approaches often pathologize individuals rather than addressing the structural and cultural conditions that produce harm [17]. Broadening and operationalizing the definition of trauma to more explicitly encompass contextual and systemic factors create space for more equitable and comprehensive school‐based practices that reflect the realities of students and educators from historically minoritized communities [15, 18, 19].

In alignment with Basch's emphasis on the links between health disparities and academic achievement [11], many of these potentially traumatic experiences are not equitably distributed across student populations. Racially and ethnically minoritized youth experience higher rates of adversity across multiple domains [20, 21]. For example, in a nationally representative sample of over 43,000 youth, Black and Latinx students were significantly more likely than their White peers to report exposure to community violence (Black: 6.9%; Latinx: 4.5%; White: 2.8%) and, unsurprisingly, racial discrimination (Black: 10.5%; Latinx: 4.4%; 1.0%) [21]. In addition, racially minoritized and newcomer (i.e., immigrant or refugee) youth are more likely to experience contextual‐level risk factors, such as adverse home, school, and/or neighborhood conditions, that increase vulnerability to bullying victimization or perpetration [22, 23, 24]. These intersecting and compounding adversities heighten the risk of socio‐emotional distress and academic disengagement, particularly through pathways such as cognitive overload, social withdrawal, and reduced connection to learning environments [11, 23, 24]. These findings underscore the need for trauma‐informed systems that explicitly attend to racialized and structural dimensions of harm.

Expanding the definition of trauma and violence exposure to encompass these diverse and systemic forms of adversity has critical implications for how schools identify and respond to student needs. Narrow definitions of risk that exclude students whose identity‐related stressors may not meet traditional clinical thresholds of trauma but nonetheless exert meaningful and chronic effects on their learning and well‐being. In contrast, more inclusive and contextually grounded definitions—such as those that recognize racialized trauma, immigration‐related stress, and other systemic or identity‐based exposures—enable schools to more accurately identify and support students.

## 2 Opportunity 2: Advance School‐Based Trauma Prevention Efforts

While a broadened definition of trauma is essential, it must be accompanied by infrastructure and practice changes that advance equitable trauma prevention in schools. To address trauma in school settings, comprehensive school mental health systems (CSMHS) offer a pathway to promoting positive mental health and well‐being for all youth, including students affected by trauma and adversity. These systems are developed through cross‐sector collaboration among leaders in education, health, and other child‐serving systems, in partnership with youth and families [25]. CSMHS offer a continuum of services across a multitiered system of support (MTSS), including universal mental health promotion/prevention (Tier 1), early intervention (Tier 2), and intensive treatment (Tier 3). When trauma‐informed and equity‐centered practices are integrated across each tier, schools are well‐positioned to prevent trauma and re‐traumatization, identify early warning signs, and provide effective responses [25].

At Tier 1, trauma‐informed approaches aim to foster safe, inclusive, and predictable school environments. Universal supports such as positive behavioral interventions and supports (PBIS) and social–emotional learning (SEL) help build relationship‐centered climates that promote resilience and reduce reliance on punitive discipline [26, 27, 28]. SEL frameworks, such as the Collaborative for Academic, Social, and Emotional Learning (CASEL) five core competencies model, have demonstrated strong effects on students' socio‐emotional skills, behavior, and academic performance, aligning closely with trauma‐informed principles of safety, connection, and empowerment [29]. A recent systematic analysis demonstrated that trauma and violence prevention efforts are effective in increasing prosocial behavior, protective factors, and academic achievement [30]. However, schools often lack the necessary infrastructure to support and sustain trauma‐informed school approaches. This can include insufficient training, ongoing support, and funding to implement evidence‐based SEL and preventive mental health programs, limited time in the school day among competing academic priorities, and difficulty tailoring curricula to reflect the needs and identities of students. Schools with the greatest need may face the scarcest resources, making it especially challenging to scale and maintain school‐wide efforts that require consistent staff buy‐in and systemic alignment.

At Tiers 2 and 3, targeted and intensive services help prevent the escalation of trauma‐related distress and support students navigating adversity that may influence their functioning, relationships, or learning. Programs such as *Cognitive Behavioral Intervention for Trauma in Schools* (CBITS) [31] and *Trauma Focused Cognitive Behavioral Therapy* (TF‐CBT) [32] are widely used to reduce trauma symptoms and promote well‐being. However, many of these models were not developed with an explicit focus on trauma that disproportionately affects racially minoritized and underserved youth—such as racial discrimination, immigration‐related stress, and community violence. Addressing health conditions that contribute to educational disparities requires intentional focus on the systemic and identity‐based factors that shape trauma exposure [15, 18]. School‐based interventions that directly focus on these forms of trauma and the inequities that compound their effects are critical. Existing culturally responsive and identity‐affirming interventions, such as *Supporting Transition Resilience of Newcomer Groups* (STRONG), *Trauma and Racism Addressed by Navigating Systemic Forms of Oppression using Resistance Methods* (TRANSFORM), and *Celebrating the Strengths of Black Youth*, show promise in supporting students whose trauma experiences are intertwined with systemic oppression [33, 34, 35]. However, schools face persistent barriers to sustaining these services, such as clinician shortages, limited access to training and supervision, and inadequate private spaces for delivery. To advance school‐based trauma prevention efforts, education, healthcare, and public health systems must collaborate to invest in culturally responsive adaptation, expand workforce capacity, and ensure sustainable funding for and implementation of interventions that reflect the lived realities of marginalized students.

## 3 Opportunity 3: Address Secondary Traumatic Stress in Trauma‐Informed Schools

Basch identified health disparities—such as unequal risk of exposure to violence and trauma—as critical barriers to academic achievement, particularly among low‐income and racially minoritized students [11]. He argued that without addressing these health challenges, educational reform efforts would be fundamentally compromised. We extend Basch's model by proposing that these same unequally distributed risk factors, particularly trauma‐related stress, must also be addressed among school personnel. One critical but often overlooked manifestation is secondary traumatic stress (STS): the trauma‐like symptoms that arise from chronic exposure to students' stories of violence, grief, and hardship [36, 37]. Many of the individuals tasked with implementing trauma‐informed practices, especially racially minoritized educators, have themselves experienced high levels of adversity, including personal trauma histories tied to systemic inequities [38, 39]. As a result, schools risk replicating a cycle of intergenerational trauma, in which adults, who once faced disproportionate exposure to risk factors as students, now struggle with STS as they support the next generation.

STS symptoms closely mirror post‐traumatic stress disorder. These symptoms impair educators' capacity to engage in relational, responsive practices that are foundational to trauma‐informed education [36]. While prevalence data specific to school personnel are limited, studies indicate that between 6% and 50% of helping professionals experience significant levels of STS [39]. Given that more than two thirds of students report exposure to at least one potentially traumatic event by age 16 [40], educators face substantial and ongoing risk of STS. This risk may be particularly elevated among racially minoritized staff, who often shoulder disproportionate emotional labor and may experience a painful resonance between students' trauma and their own lived experiences [38]. Several studies have found that educators with personal trauma histories are more likely to report symptoms of STS, and racially minoritized educators are more likely to report such histories due to persistent structural and social inequities [38, 39]. Educators experiencing STS are more likely to disengage from students, reduce practice effectiveness, and, ultimately, leave the profession [41, 42].

Although educator stress and STS are acknowledged in established trauma‐informed school frameworks [43], these components are often underemphasized in actual practice. Many implementation efforts continue to focus primarily on student trauma, with limited attention to equipping school personnel to recognize and respond to their own trauma exposure. This imbalance often reflects systemic pressures on schools to demonstrate student outcomes, coupled with limited resources, time, and training devoted to staff wellness. This disconnect between framework and practice threatens not only staff well‐being and retention but also undermines the broader goals of trauma‐informed care. Elevating STS as a central concern affirms that educator wellness is foundational to the success and sustainability of trauma‐informed systems in schools.

## 4 Implications for School Health Research

Future research should examine how trauma‐informed practices can be implemented equitably across diverse school contexts, particularly those serving racially minoritized and underserved youth. Additional research is needed to evaluate how schools adapt trauma‐informed interventions to address identity‐based stressors and to assess whether these adaptations lead to improved health and educational outcomes. Research should also explore how schools operationalize cultural responsiveness in trauma‐informed systems and the extent to which these efforts are reflected in policy, practice, and professional development. In addition, more attention is needed to the implementation of conditions that support or hinder sustainable, equity‐centered approaches. This includes studying workforce development models, cross‐sector collaboration strategies, and structural supports for educator wellness and STS prevention. Finally, elevating youth, family, and educator voices in school health research is critical to ensuring that trauma‐informed approaches are sustainable, contextually relevant, culturally affirming, and informed by lived experience.

## 5 Discussion

### 5.1 Implications for School Health Policy, Practice, and Equity

The National Child Traumatic Stress Network offers a public health‐oriented framework that can guide schools in building trauma‐informed systems aligned with equity goals [43]. Its emphasis on cultural responsiveness, school‐community partnerships, and centering the voices of youth and families underscores the importance of embedding trauma‐informed care into broader efforts to address systemic disparities. The model encourages schools to review policies that contribute to unequal outcomes, adapt interventions to reflect students' cultural identities, and prioritize staff wellness and capacity‐building as core components of sustainable implementation. Schools can leverage this framework to align trauma‐informed practices with local needs, ensure interventions are contextually relevant, and strengthen infrastructure through cross‐sector collaboration.

Implementation efforts must move beyond isolated strategies to build integrated, equity‐centered systems that respond to the full complexity of trauma and violence in schools. A foundational step is to broaden the school‐based definitions of potentially traumatic experiences to include chronic and identity‐based adversities, such as racial discrimination, bullying, and immigration‐related stress. To act on this more inclusive definition, schools need a clear understanding of the specific adversities their students face and the resources available to address them. As such, local education agencies (LEAs, e.g., school districts) should consider comprehensive needs assessments to illuminate the scope and context of trauma within their school communities, surfacing concerns like exposure to community violence, grief and loss, or racial discrimination that may not be visible through academic or behavioral data alone [44]. This also includes attention to staff experiences of STS and related workforce strain. These assessments are most effective when paired with resource mapping, a process that inventories current programs, personnel, and services across tiers of support. Together, these tools help LEAs identify gaps in care and areas where existing efforts can be aligned with trauma‐informed goals. To ensure equity, data collected through needs assessments and resource mapping should be disaggregated by race, ethnicity, language, and other relevant demographics. This allows LEAs to determine whether national patterns of disparity are reflected in their own context—and to tailor interventions accordingly. When implemented with fidelity, this approach lays the groundwork for trauma‐informed systems that are not only responsive, but also equitable and sustainable.

Similarly, professional development for school staff should be inclusive of a broad range of trauma exposures. Effective professional development should highlight both the shared and distinct features of different potentially traumatic experiences and offer culturally responsive strategies for addressing them in the classroom and through school‐wide practices. One exemplar is the Healthy Environments and Response to Trauma in Schools (HEARTS) program, developed by the University of California, San Francisco [45]. HEARTS offers a multitiered approach that supports schools in becoming trauma‐informed and healing‐centered. The program combines training in trauma neurobiology with practical strategies to shift school culture, build staff capacity, and improve student outcomes. A key strength of HEARTS is its explicit integration of racial equity and cultural responsiveness, recognizing that trauma does not occur in a vacuum but is often shaped by systemic oppression and identity‐based stress. Through ongoing professional development, consultation, and coaching, HEARTS equips school personnel to cultivate environments that are not only safe and supportive but also affirming and inclusive for all students—especially those from historically marginalized backgrounds [45].

LEAs should build and sustain cross‐sector, equity‐focused partnerships to codesign, implement, and evaluate trauma‐informed strategies that can be tailored by individual schools. Addressing the complexity of trauma, particularly when it intersects with systemic inequities, requires collaboration beyond school walls. Partnerships with youth, families, educators, mental health professionals, pediatric clinicians, community organizations, and cultural leaders ensure that trauma‐informed approaches are grounded in lived experience and attuned to local contexts. These collaborations help schools avoid top‐down solutions that may miss key community priorities or inadvertently replicate harm. These partnerships are essential for expanding access to student mental health supports and for addressing workforce challenges related to STS, burnout, and retention. Community‐based organizations can help fill service gaps for students while also serving as partners in providing consultation, supervision, and wellness supports for school staff. In addition, cross‐sector teams are particularly valuable in facilitating data‐driven decision‐making. For example, partners can help interpret needs assessment data, identify culturally responsive practices, and ensure that action plans reflect the strengths and needs of historically marginalized populations. Community‐based organizations can also help fill service gaps, expand access to mental health supports, and offer culturally affirming resources that schools and LEAs may not have in‐house. Involving students and families in ongoing planning and evaluation strengthens relevance and accountability and helps build relational trust—a key driver of sustained systems change. These partnerships are also critical for navigating the structural barriers that often hinder implementation. For example, when schools lack the internal capacity to provide trauma‐specific interventions or support staff wellness, community agencies can serve as vital collaborators. By coordinating across systems, LEAs can braid funding streams, align goals, and create more seamless pathways for support. When done well, these collaborative efforts enhance both the infrastructure and culture of trauma‐informed schools, ensuring that change is sustainable.

In addition, to create sustainable, healing‐centered school environments, LEAs must embed policies and practices that actively promote educator wellness and prevent STS. This includes investing in universal supports, which may include strategies such as designated wellness spaces, restorative staff practices, and flexible scheduling policies that allow for emotional recovery. Targeted supports might involve affinity groups, reflective supervision, or peer consultation structures that help normalize and process emotional labor—particularly for staff navigating trauma that mirrors their students' experiences. Intensive supports must include access to confidential, culturally responsive mental health services, ideally offered in partnership with community providers.

Finally, although these approaches are critical for advancing equitable and trauma‐responsive systems, implementation may be constrained by variability in school readiness, resources, and stakeholder buy‐in. Schools operating in contexts with limited capacity may benefit from incremental strategies, such as integrating trauma‐responsive and culturally responsive practices into existing initiatives, leveraging current staff roles and partnerships, and prioritizing foundational elements (e.g., relationship‐building, safe and supportive environments) as initial steps toward broader systems change.

## 6 Conclusions

Building on Basch's foundational recognition that health disparities are central to educational inequities [11], this paper highlights critical opportunities to evolve and strengthen school‐based trauma prevention and intervention. In the years since Basch's call to action, schools have made meaningful progress in adopting trauma‐informed approaches and prioritizing student well‐being. The next phase of this work requires a broader, more systemic response—one that recognizes the full scope of trauma, including identity‐based and structural stressors; leverages comprehensive school mental health frameworks to build sustainable infrastructure; and positions educator well‐being, particularly in relation to STS, as essential to implementation. Together, these strategies advance a vision of trauma‐informed schools that are not only safe and supportive, but also equity‐centered, culturally responsive, and sustainable.

## Funding

This work was supported by Johns Hopkins Bloomberg School of Public Health.

## Ethics Statement

The authors have nothing to report.

## Conflicts of Interest

The authors declare no conflicts of interest.

## Data Availability

Data sharing not applicable to this article as no datasets were generated or analysed during the current study.
